# miR-30a targets *STOX2* to increase cell proliferation and metastasis in hydatidiform moles via ERK, AKT, and P38 signaling pathways

**DOI:** 10.1186/s12935-022-02503-3

**Published:** 2022-03-04

**Authors:** Zhenzhen Guo, Chenyu Zhu, Youhui Wang, Zhen Li, Lu Wang, Jianhui Fan, Yuefei Xu, Na Zou, Ying Kong, Dong Li, Linlin Sui

**Affiliations:** 1 Core Lab Glycobiol & Glycoengn, College of Basic Medical Sciences, Dalian Medical University, Dalian, 116044 Liaoning China; 2 Department of Pathology, Dalian Municipal Women And Children’s Medical Center, Dalian, 116044 Liaoning People’s Republic of China

**Keywords:** miR-30a, STOX2, Hydatidiform mole, ERK, AKT

## Abstract

**Background:**

A hydatidiform mole is a condition caused by abnormal proliferation of trophoblastic cells. MicroRNA miR-30a acts as a tumor suppressor gene in most tumors and participates in the development of various cancers. However, its role in hydatidiform moles is not clear.

**Methods:**

Quantitative real-time reverse transcription PCR was used to verify the expression level of miR-30a and *STOX2* (encoding storkhead box 2). Flow cytometry assays were performed to detect the cell cycle in cell with different expression levels of miR-30a and *STOX2*. Cell Cycle Kit-8, 5-ethynyl-2′-deoxyuridine, and colony formation assays were used to detect cell proliferation and viability. Transwell assays was used to test cell invasion and migration. Dual-luciferase reporter assays and western blotting were used to investigate the potential mechanisms involved.

**Result:**

Low miR-30a expression promoted the proliferation, migration, and invasion of trophoblastic cells (JAR and HTR-8). Dual luciferase assays confirmed that *STOX2* is a target of miR-30a and resisted the effect of upregulated miR-30a in trophoblastic cells. In addition, downregulation of *STOX2* by miR-30a could activate ERK, AKT, and P38 signaling pathways. These results revealed a new mechanism by which ERK, AKT, and P38 activation by miR-30a/STOX2 results in excessive proliferation of trophoblast cells in the hydatidiform mole.

**Conclusions:**

In this study, we found that miR-30a plays an important role in the development of the hydatidiform mole. Our findings indicate that miR-30a might promote the malignant transformation of human trophoblastic cells by regulating *STOX2*, which strengthens our understanding of the role of miR-30a in regulating trophoblastic cell transformation.

**Supplementary Information:**

The online version contains supplementary material available at 10.1186/s12935-022-02503-3.

## Background

Gestational trophoblastic disease (GTD) refers to a group of placental trophoblast diseases characterized by abnormal proliferation [1], including the hydatidiform mole, villus cancer, epithelial itch trophoblastic tumors, and placental site trophoblastic tumors [2–4]. GTD can be divided into two types: Benign and malignant, among which the hydatidiform mole is the only benign GTD; the others are malignant. Choriocarcinoma can be caused by a hydatidiform mole, an ectopic pregnancy, and abortion [5]. A hydatidiform mole is an abnormal pregnancy characterized by placental villus edema and abnormal growth of trophoblastic cells [6]. Placental edema can lead to a series of pathological phenomena, such as edema abortion (HA), partial hydatidiform moles (PHMs), and complete hydatidiform moles (CHMs). The occurrence of hydatidiform moles accounts for 80% of GTD [7], and the incidence varies in different regions of the world [8, 9]. There are many factors that induce hydatidiform moles, such as age, ethnicity, genetics, spontaneous abortion, and nutritional restriction [10]. The incidence of hydatidiform moles in women between the ages of 21–35 years is lower than that in women over the age of 35 or under the age of 21 [11]. Compared with the general female population, women who have a history of spontaneous abortion are 2–3 times more likely to have a hydatidiform mole [12]; women who have had a hydatidiform mole are 10–20 times more likely to develop hydatidiform moles in subsequent pregnancies compared with women who have not; and about 20% have the possibility of malignant transformation after resection. However, the pathogenesis of the hydatidiform mole is currently unclear.

MicroRNAs (miRNAs) regulate gene expression by binding to the 3′ untranslated region (UTR) of their target gene mRNA, acting as negative regulatory factors [13]. There is increasing evidence that miRNAs play an important role in the pathogenesis and progression of various tumors by regulating cell proliferation [14], the cell cycle [15], inflammatory responses [16], cell differentiation [17], apoptosis, and metastasis [18]. miRNAs also play a regulatory role during embryonic development [19]. The miRNA-518 family is a specific biomarker of the placenta. miR-518b, which is abnormally expressed in placental tissues during preeclampsia, not only regulates early growth response 1 (EGR1)-mediated angiogenesis and migration of trophoblast cells, but also regulates the establishment of the hypoxia model of early embryonic development [20]. Previous studies have shown that mir-30a-5p is located in the 6q13 region of chromosome 6 and is dysregulated in certain tumors [21]. In HEPG2 and MHC97l cancer cells, overexpression of mir-30a completely blocked the activation of the KRAS proto-oncogene, GTPase (KRAS)/ Raf-1 proto-oncogene, serine/threonine kinase (c-RAF)/MAPK/ERK kinase (MEK)/extracellular regulated kinase (ERK) pathway. These findings suggested that mir-30a plays a role in the growth, apoptosis and metastasis of hepatoma cells by regulating the k-RAS/c-RAF/MEK/ ERK signaling pathway, and might become a targeted biomarker for liver cancer treatment [22]. miR-30a is overexpressed in the placenta of patients with eclampsia, and might exert its effect by influencing the invasion and apoptosis of trophoblast cells by targeting *IGF1* (encoding insulin like growth factor 1) [23]. Storkhead box 2 is a winged helix domain protein, encoded by the *STOX2* gene on chromosome 4q35, near the chromosomal region associated with preeclampsia. STOX2 is a transcription factor involved in trophoblast differentiation and is the most important collateral of STOX1 [24, 25]. The abnormal expression of STOX2 in neural crest stem cells and lung cells of the offspring of asthmatic inflammatory model mice was analyzed by transcription expression [26]. In addition, compared with non-pregnant mice, the inflammatory response of STOX2 to air pollutants in pregnant mice was increased [27]. Melanoma suppressor protein (MIA) affects the expression of STOX2 in a paracrine manner, promoting the proliferation and metastasis of oral squamous cell carcinoma [28]. The same study found that STOX2 combined with anticancer drugs, such as paclitaxel, cisplatin, or 5-FU, could reduce the drug resistance of cancer cells, providing a new treatment paradigm [28]. In our previous study [29], we compared the expression levels of *STOX1* and *STOX2* in decidual tissue from pregnancies with pre-eclampsia and/or fetal growth restriction (FGR), and found that *STOX1* did not show differential gene expression between any of the groups, while the expression of *STOX2* in the decidua of pregnancies with preeclampsia and FGR was significantly lower than that in the control group.

Our laboratory has been committed to the study of the pathogenesis of hydatidiform moles. Previously, we detected the expression of miRNAs between hydatidiform moles and normal villi (results unpublished) and identified a large number of differentially expressed miRNAs. Among them, miR-30a was the miRNA with the largest differential multiple. Therefore, we further investigated miR-30a and found that it had low expression in hydatidiform mole tissue [30]. In addition, we found that miR-30a can affect the occurrence of hydatidiform moles by regulating UDP-GlcNAc:betaGal beta-1,3-N-acetylglucosaminyltransferase 5 (B3GNT5). Thus, the regulatory mechanism of miR-30a appears to be multifaceted, and whether it regulates other genes and affects other pathways or the cell cycle is unknown. Further study at our laboratory showed that another miRNA, miR-196b, inhibits cell migration and invasion through targeting *MAP3K1* (encoding mitogen-activated protein kinase kinase kinase 1) in hydatidiform moles [31]. Determining the molecular pathogenesis of the hydatidiform mole will contribute to the treatment and prevention of hydatidiform moles. In the present study, based on the observation of the low expression of miR-30a in hydatidiform mole tissue, we combined laboratory database and biological software prediction to identify *STOX2* as a possible target of miR-30a. Further experiments showed that miR-30a, by regulating *STOX2*, actives the protein kinase B (AKT), ERK, and P38 signaling pathways to affect the development of hydatidiform moles.

## Methods

### Sample collection

Twenty formalin fixed, paraffin embedded hydatidiform mole tissues, 10 fresh hydatidiform mole tissues, and 15 normal placental tissues were collected from the Dalian Women's and Children's Hospital. The use of these samples and the experimental protocol were approved by the ethics committees of Dalian Medical University. All patients provided written informed consent.

### Cell culture and transfection

The human trophoblast cell line JAR and the human cervical cancer cell line HeLa were purchased from the American Type Culture Collection (Manassas, VA, USA). HTR-8/SVneo cells were obtained from the Animal Institute of the Chinese Academy of Sciences (Beijing, China). The cells were maintained in Roswell Park Memorial Institute (RPMI-1640 medium (Invitrogen; Thermo Fisher Scientific, Waltham, MA, USA) containing 10% fetal bovine serum (ScienCell, Carlsbad, CA USA), 1% penicillin–streptomycin solution (Thermo Fisher Scientific). Cultures were maintained in a cell incubator with a humidified atmosphere of 5% CO_2_ at 37 °C.

The miR-30a mimics and mimic negative control (RiboBio, Guangzhou, China), and the miR-30a inhibitor and inhibitor negative control were purchased from Guangzhou RiboBio Co., Ltd, (Guangzhou, China). Small interfering RNA (siRNA) targeting *STOX2* and the negative control (NC) siRNA were produced by Shanghai GenePharma (Shanghai, China). The si-STOX2 sequence was 5′-AUGGGAGACAUACUGAUGGTT-3′ and the si-NC sequence was 5′-ACGUGACACGUUCGGAGAATT -3′. The *STOX2* cDNA and corresponding negative control were constructed by GeneCopoeia Inc. (Rockville, MD, USA). Cells were seeded in 6-well plates, grown to 70–80% confluence, and transfected with the various constructs and vectors using the Lipofectamine® 2000 reagent according to the manufacturer's instructions.

### Quantitative real-time reverse transcription PCR (qRT-PCR)

Total RNAs were extracted from JAR and HTR-8 cells using the TRIzol reagent. cDNA was synthesized from the RNA using a TransScript All-in-one First-Strand cDNA Synthesis SuperMix for qPCR kit (One-Step gDNA Removal) (TransGen, Beijing, China) according to the manufacturer’s specifications. The DNA was then used as the template for qPCR using a TransStart Top Green qPCR SuperMix (TransGen), which was analyzed on an ABI 7500 Real-Time PCR System (Applied Biosystems; Foster City, CA, USA). The qPCR conditions were 94 °C for 30 s; followed by 40 cycles of denaturation at 94 °C for 5 s and annealing/elongation at 60 °C for 30 s. U6 was used as the internal reference for miR-30a, and *GAPDH* (encoding glyceraldehyde-3-phosphate dehydrogenase) was used as the control for *STOX2* expression. The qPCR primer were as follows: *STOX2* forward: 5′-AGCCTGTCCCTCCTCAAATCTCA-3′, reverse: 5′-CTCTGTGTTCTTGTTTGCCCCT-3′; *GAPDH* forward: 5′- GTGAAGGTCGGAGTCAACG-3′, reverse: 5′-TGAGGTCAATGAAGGGGTC-3′; Relative expression was calculated using the 2^−ΔΔct^ method. All experiments were performed on triplicate samples.

### Cell Counting Kit-8 (CCK-8) assay

Transfected Cells were seeded in 96-well plates in 200 μl of medium at a density of 5000 cells per well and cultured for 24, 48, and 72 h. CCK-8 reagent (Dojindo, Kumamoto, Japan) was added into each well. After incubation for 4 h at 37 °C, the absorbance at 450 nm was detected using a microplate reader (BioTek, Winooski, VT, USA).

### Colony formation assay

At 48 h after transfection, 3 × 10^3^ Cells were seeded in the wells of 6-well plates and incubated for 15 days. The colonies formed were fixed with methanol for 30 min and stained with 1% crystal violet for 15 min. Cell numbers was counted under a microscope using Image J software (NIH, Bethesda, MD, USA).

### 5-ethynyl-2'-deoxyuridine (EdU) assay

The EdU reagent (Beyotime, Shanghai, China) was added into wells of 96-well plates containing transfected cells and cultured for 2 h in a 37 °C incubator. The cells were then fixed with 4% paraformaldehyde for 30 min, incubated with 0.1% Triton-X 100 for 15 min, and then with with Azide-488 for 30 min at room temperature in the dark. Images were taken under an inverted microscope (Olympus, Tokyo, Japan).

### Transwell assay

Transwell plates (8 μm pore size, 24-wells; Corning Inc., Corning, NY, USA) were used to evaluate the migratory and invasive potential of JAR and HTR-8 cells. For the migration assay, transfected cells were collected and resuspended in 1 ml of serum-free RPMI-1640 medium, and 5 × 10^4^ cells were added to the upper chamber of the Transwell apparatus; RPMI-1640 medium with 10% FBS was seeded into the lower chamber. After incubation for 24 h in 5% CO_2_ at 37 °C, the migratory cells were fixed with methanol for 30 min and stained with 0.2% crystal violet for 30 min. For the invasion assay, the upper Transwell chamber was pre-coated with Matrigel (BD Biosciences, San Jose, CA, USA), 5 × 10^4^ transfected cells were seeded into the upper chamber in serum-free RPMI-1640 medium, after incubation for 30 h, the cells that had not invaded were wiped off with a cotton swab, and the invasive cells were fixed with methanol for 30 min and stained with 0.2% crystal violet for 30 min. The cells in all the migratory and invasive chambers were counted under a light microscope (Olympus).

### Western blotting assay

After the cells were transfected for 48 h, total protein was extracted using a ProteinExt® Mammalian Total Protein Extraction Kit (TransGen), and the amount of protein was determined using the bicinchoninic acid (BCA) method (TransGen). Protein (30 or 60 µg) was separated using 10% sodium dodecyl sulfate–polyacrylamide gel electrophoresis (SDS-PAGE) and transferred onto a nitrocellulose membrane (Millipore, Bedford, MA, USA) using cold transfer buffer. The membrane was stained with Ponceau S (Beyotime, Shanghai, China) and washed with Tris buffered saline-Tween 20 (TBST). The membrane was blocked in 5% non-fat milk for 2 h at room temperature. The membrane was then incubated with primary antibodies overnight 4 °C. The primary antibodies used were as follows: anti-STOX2 (1:1000, Abcam, Cambridge, MA, USA), anti-ERK (1:1000, Beyotime), anti-phospho(p)-ERK (1:500, Beyotime), anti-AKT (1:1000, Beyotime), anti-p-AKT (1:500, Abcam), anti-P38 (1:1000, Elabscience, Wuhan, China), anti-p-P38 (1:500, Elabscience), and anti-GAPDH (1:4000, Proteintech, Rosemont, IL, USA). Next day, the membrane was washed and then incubated with the secondary antibody at room temperature for 1 h. The blots were then developed by chemiluminescence using Pierce ECL kits (Pierce Biotechnology, Rockford, IL, USA). The gray values of the immunoreactive protein bands were analyzed using Image J software.

### Luciferase activity assay

miRNA prediction websites (miRBase, TargetScan, and PicTar) was used to predict the binding site of miR-30a in the *STOX2* 3′ UTR. The 3′-UTR of *STOX2* was synthesized by PCR and cloned into the Xhol site downstream of the Renilla luciferase gene in the PmiR reporter vector (Promega, Madison, WI, USA). The wild-type (WT) or mutated (Mut) miR-30a seed sequences in the 3′ UTR of *STOX2* were constructed onto the PmiR reporter vector. HeLa cells were seeded into 12-well plates and cotransfected with miR-30a mimics or negative control and the WT or Mut vector. After incubation for 24 h, a Dual-Luciferase® Reporter Assay was carried out according to the manufacturer’s manual (Promega). The luciferase activities were measured using a Fluorescence/Multi-Detection Microplate Reader (BioTek).

### Cell cycle analysis

At 48 h after transient transfection, cells was collected and washed with phosphate-buffered saline twice, and then fixed using cool 70% ethanol overnight at 4 °C. The fixed cells were stained with propidium iodide (PI)/RNase solution (Sungene, Tianjin, China) for 30 min at room temperature and analyzed using a FACS Calibur flow cytometer (BD Biosciences). The percentage of cells in each phase of the cell cycle was analyzed using the ModFit software (Verity Software House, Topsham, ME, USA.

### Immunohistochemistry (IHC)

Tissue sections were dewaxed in xylene and dehydrated using an ethanol gradient. The activity of endogenous peroxidase was blocked using 3% H_2_O_2_ for 20 min in the dark. Goat serum was added onto the tissues using a dropwise method over 20 min at room temperature. Then, the sections were incubated with the following primary antibodies overnight at 4 °C: anti-STOX2 (1:100, Abcam), anti-AKT (1:70, Beyotime), anti-ERK (1:100, Beyotime), anti-p-ERK (1:50, Beyotime), anti-p-AKT (1:70, Abcam), anti-P38 (1:100, Elabscience), and anti-p-P38 (1:50, Elabscience). Next, the secondary antibody and horseradish peroxidase streptavidin were incubated for 30 min at 37 °C, respectively. The sections were stained using 3, 3'-diaminobenzidine (DAB) (OriGene Technologies, Beijing, China) and hematoxylin (KeyGEN BioTECH, Jiangsu, China). Tissues sections were imaged under a light microscope.

### Hematoxylin and eosin (HE) staining

The Hydatidiform mole and normal placenta tissues were fixed in 4% formaldehyde and embedded in paraffin for Hematoxylin and eosin (HE) staining. The slices were stained with hematoxylin for 20 min and eosin with for 30 s to 1 min. The sections were then analyzed under a light microscope.

### Statistical analysis

All data are presented as mean ± SD and were analyzed using GraphPad Prism 6.0 software (GraphPad Inc., La Jolla, CA, USA). All experiments were repeated three times independently. The significance of the difference between two groups was assessed via one-way analysis of variance (ANOVA) and a P value < 0.05 was considered statistically significant.

## Result

### Upregulation of miR-30a inhibited trophoblastic cell proliferation, cell cycle, and metastasis

**Fig. 1:**
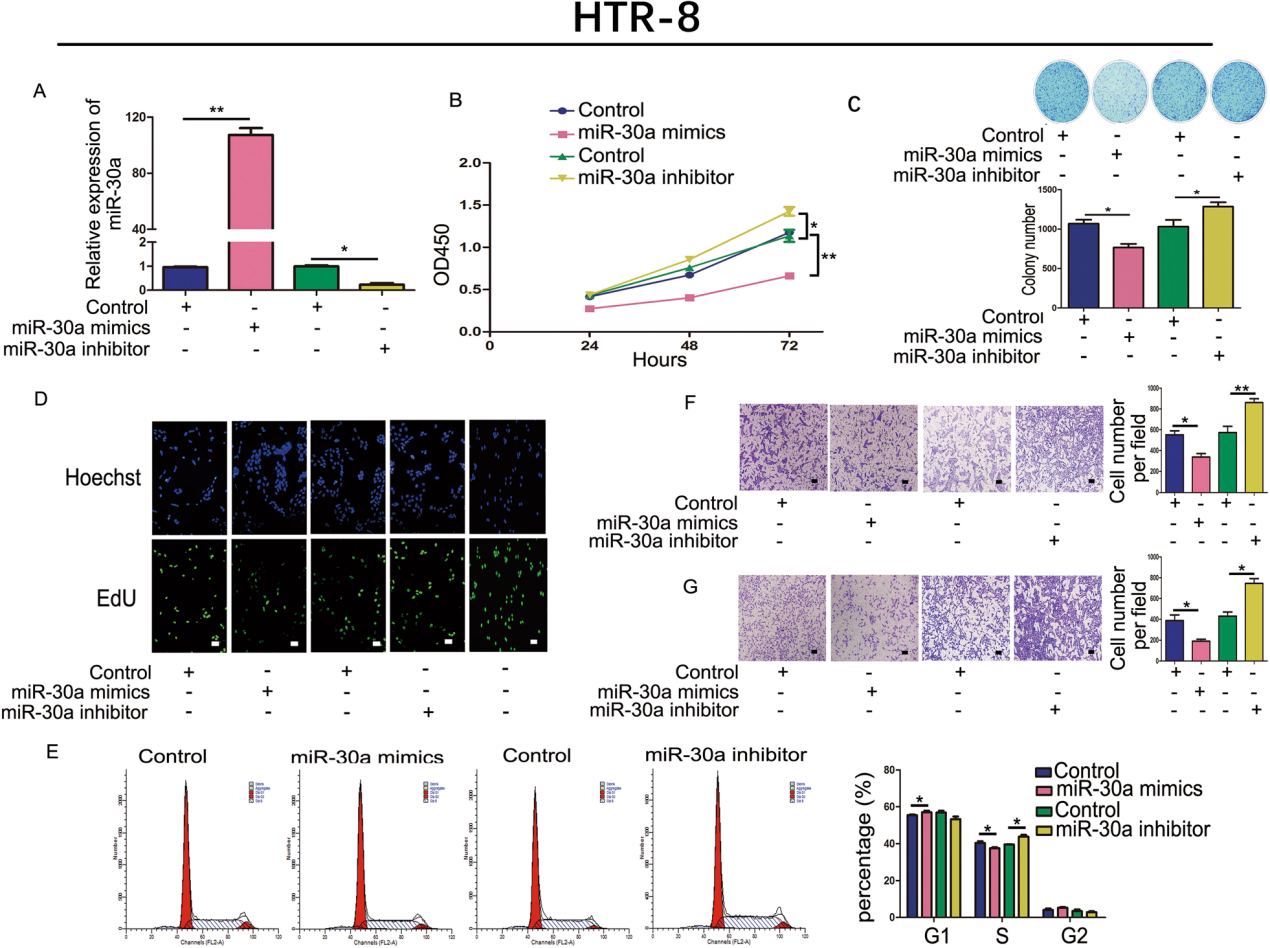
Upregulation of miR-30a inhibited the proliferation, cell cycle, and metastasis of HTR-8 cells. **A** The level of miR-30a as detected using qRT-PCR. **B** CCK-8 assay to test the proliferation of HTR-8 cells transfected with miR-30a mimics/negative control or miR-30a inhibitor/negative control. **C** Colony formation ability of the different groups of cells. **D** EdU assay to detect the growth ability of HTR-8 cells expressing different levels of miR-30a. The last line is the positive control. **E** Flow cytometry analysis of the cell cycle. The migration (**F**) and invasion (**G**) of HTR-8 cells analyzed using Transwell assays. Bar = 100 μm. *P < 0.05, **P < 0.01

In our previous study, we demonstrated that the expression level of miR-30a was lower in hydatidiform moles [30]. However, the regulatory mechanism of miR-30a in hydatidiform moles is still unclear. In this study, we explored the mechanism of miR-30a in hydatidiform mole disease at the cellular level. HTR-8 cells were transfected with miR-30a mimics/negative control and miR-30a inhibitor/negative control and the transfection efficiency was confirmed (Fig. 1A). A CCK-8 assay showed that the proliferation of HTR-8 cells was weakened by upregulating miR-30a; conversely, suppression of miR-30a increased cell proliferation (Fig. 1B). Transfection with miR-30a mimics reduced the colony formation ability of HTR-8 cells (Fig. 1C and Additional file 1: Fig. S1). Compared with cells transfected with the negative control, the fluorescence activity of cells transfected miR-30a mimics decreased in the EdU assay. This suggested that cell proliferation was inhibited (Fig. 1D). Furthermore, we demonstrated that downregulation miR-30a enhanced the proportion of cells S-phase of the cell cycle, thus promoting the growth of trophoblastic cells (Fig. 1E and Additional file 1: Fig. S1B). Transwell assays for migration and invasion of HTR-8 cells showed that compared with cells transfected with the negative control, cells transfected the miR-30a mimics had impaired migration and invasion abilities, whereas downregulating miR-30a increased the migration and invasion abilities of HTR-8 cells (Fig. 1F, G). These results demonstrated that miR-30a inhibited cell viability, the cell cycle, and metastasis of trophoblastic cells.

### The effect of different expression levels of *STOX2* on the proliferation and metastasis of trophoblastic cells

**Fig. 2:**
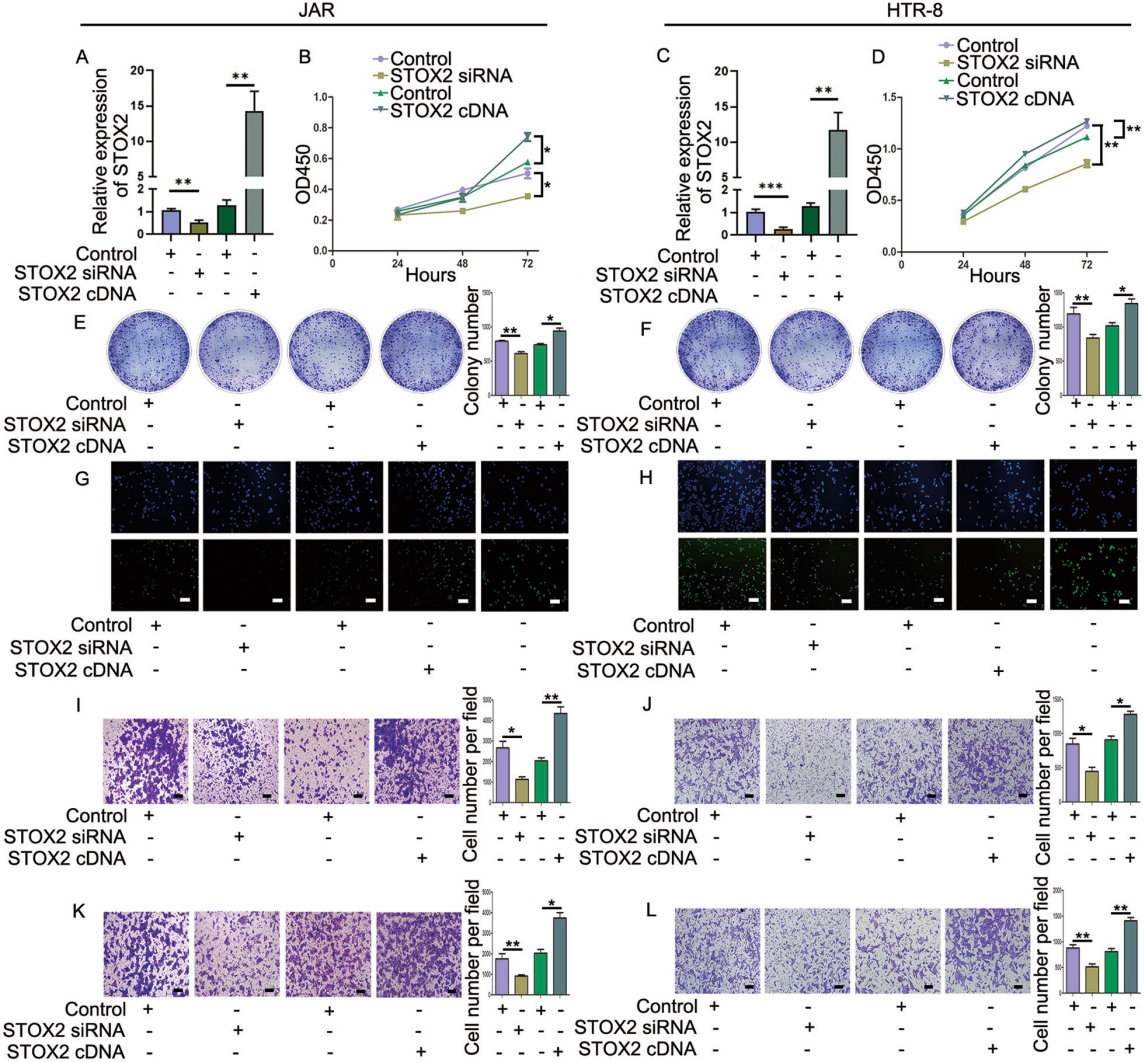
The effect of different expression levels of STOX2 on the proliferation and metastasis of trophoblastic cells. **A** and **C** Relative STOX2 expression was detected using qRT-PCR in JAR and HTR-8 cells transfected with *STOX2* siRNA/negative control or *STOX2* cDNA/negative control. **B** and **D** The effect of different STOX2 levels on JAR and HTR-8 cell proliferation in each group. **E** and **F** Colony formation ability of cells expressing different levels of STOX2. **G** and **H** Experiment of Edu in JAR and HTR-8 cells transfected with *STOX2* siRNA or *STOX2* cDNA. The last line is the positive control. Transwell assay migration (**I** and **J**) and invasion (**K** and **L**) abilities of the different groups of cells. Bar = 100 μm. *P < 0.05, **P < 0.01

To investigate the effect of STOX2 on the proliferation and metastasis of trophoblast cells, firstly, we tested the transfection efficiency of STOX2 siRNA or the *STOX2* cDNA construct using qRT-PCR, which confirmed the expected reduced and increased expression of *STOX2*, respectively (Fig. 2A, C). We performed CCK-8, colony formation, and EdU assays in JAR and HTR-8 cells. The CCK-8 assay showed that upregulation of *STOX2* promoted cell proliferation significantly compared with that in the control group, whereas the proliferation ability of the cells was significantly decreased following downregulation of *STOX2* (Fig. 2B, D). The ability of cells transfected with the *STOX2* cDNA construct to form colonies increased; however, the colony forming ability of cells in the *STOX2* siRNA group decreased significantly (Fig. 2E, F). Similarly, the EdU assay demonstrated that the fluorescence activity (cell replication) was enhanced after upregulating *STOX2*, but decreased after silencing *STOX2* (Fig. 2G, H).

Transwell assays revealed that overexpression of *STOX2* enhanced cell migration and invasion, whereas silencing of STOX2 reduced cell migration and invasion (Fig. 2I–L). These results indicated that upregulation of STOX2 promoted the metastatic behavior of trophoblastic cells.

### miR-30a targets the 3′ UTR of STOX2 mRNA and has low expression in hydatidiform moles.

**Fig. 3:**
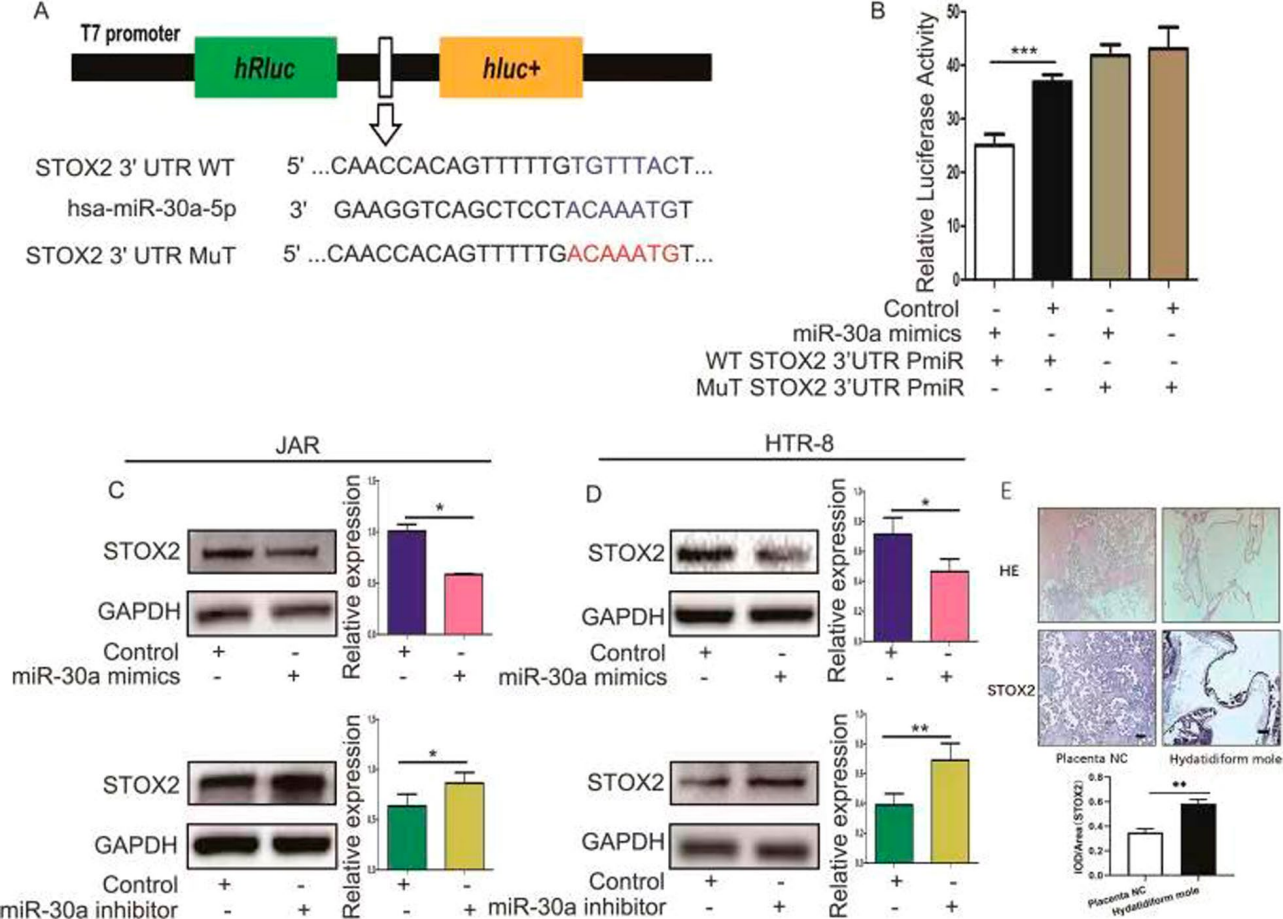
miR-30a targets the 3′-UTR of the *STOX2* mRNA. **A** Schematic showing the position of the cloned sequences in the luciferase reporter construct. Below are shown the *STOX2* 3′-UTR region containing the miR-30a-5p binding site, the miR-30a-5p sequence, and the mutated binding site, used to construct the wild-type and mutant vectors, respectively. **B** HeLa cells were transfected with wild-type or mutant luciferase reporter vectors with miR-30a mimics to determine the luciferase activity. **C** and **D** Western blotting assay to detect the protein levels of STOX2 after regulating of miR-30a expression using mimics or inhibitors. **E** IHC showing the expression of STOX2 in hydatidiform mole tissues and normal placenta. Bar = 100 μm. *P < 0 .05 or **P < 0 .01, ***P < 0.001

Bioinformatic analysis (miRBase, TargetScan, PicTar) predicted *STOX2* as a candidate target gene for miR-30a. As shown in Fig. 3A, an miR-30a binding site was identified in the 3′ UTR region of *STOX2*. Subsequently, we cloned the WT and Mut 3′ UTR of *STOX2* into a luciferase reporter vector, which contained the Renilla luciferase gene fused to the *STOX2* 3′ UTR sequence and expressed firefly luciferase for normalization. These vectors were transfected into HeLa cells with miR-30a mimics or their negative controls. As expected, compared with co-transfecting WT STOX2 3′UTR PmiR vector and the negative control, the luciferase reporter activity in cells was reduced after co-transfection WT STOX2 3′ UTR PmiR and miR-30a mimics; however, the luciferase reporter activity was unchanged when we co-transfected Mut STOX2 3′ UTR PmiR and miR-30a mimics or the negative control (Fig. 3B). Furthermore, western blotting showed that the protein level of STOX2 was reduced in JAR and HTR-8 cells transfected with miR-30a mimics. Conversely, when we inhibited the expression of miR-30a, the STOX2 protein level increased (Fig. 3C, D). In our previous research, we detected that miR-30a expression was lower in hydatidiform mole tissues than in normal placenta tissues [30]. In the present study, we found that the expression of STOX2 was high in hydatidiform mole tissues (Fig. 3E). In addition, there was a correlation between the expression of miR-30a and STOX2 (Additional file 2: Fig. S2). These results confirmed our prediction that *STOX2* is a target of miR-30a and that miR-30a might participate in the pathogenesis of hydatidiform moles by targeting *STOX2*.

### STOX2 mitigates miR-30a inhibition of proliferation and metastasis in trophoblastic cells

**Fig. 4:**
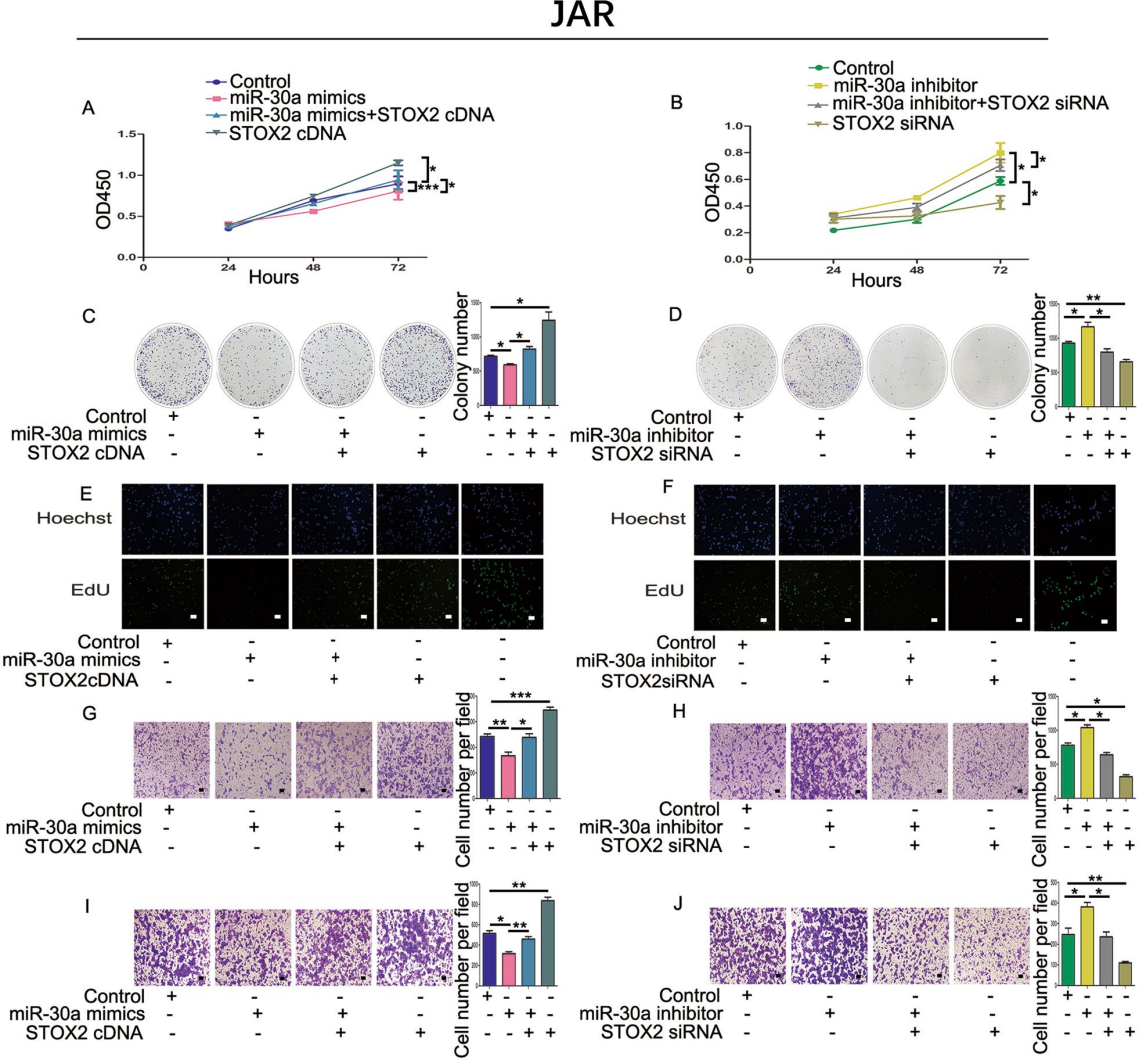
STOX2 mitigates miR-30a stimulation of proliferation and metastasis in JAR cells. **A** and **B** CCK-8 assay showed the proliferation of JAR cells co-transfected with miR-30a mimics and *STOX2* cDNA or miR-30a inhibitor and *STOX2* siRNA. **C** and **D** Colony formation experiment assessing the growth of JAR cells. **E** and **F** The viability of JAR cells was analyzed using an EdU assay. The last line is the positive control. **G** and **H** Cell migration detected after co-transfection with *STOX2* cDNA or miR-30a inhibitor and *STOX2* siRNA into JAR cells. **I** and **J** Cell invasion detected after co-transfection with *STOX2* cDNA or miR-30a inhibitor and *STOX2* siRNA into JAR cells. Bar = 100 μm. *P < 0 .05 or **P < 0 .01, ***P < 0.001

**Fig. 5:**
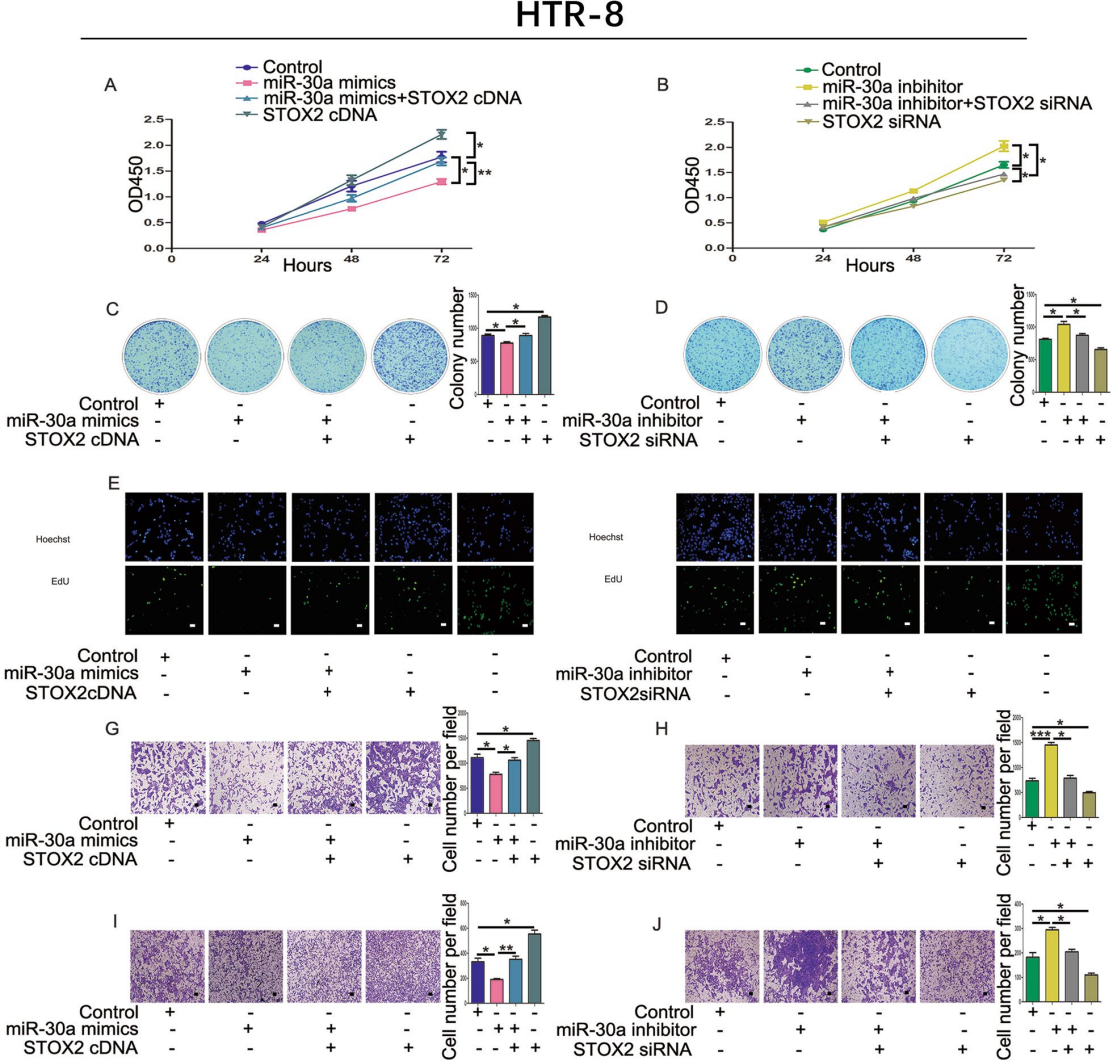
STOX2 mitigates miR-30a stimulation of proliferation and metastasis in HTR-8 cells. **A** and **B** CCK-8 assay showing the proliferation of HTR-8 cells co-transfected with miR-30a mimics and *STOX2* cDNA, or with the miR-30a inhibitor and *STOX2* siRNA. **C** and **D** Colony formation assay for the growth of HTR-8 cells. **E** and **F** The viability of HTR-8 cells was analyzed using an EdU assay. The last line is the positive control. **G** and **H** Cell migration assessed after co-transfection of the *STOX2* cDNA or miR-30a inhibitor and *STOX2* siRNA into HTR-8 cells. **I** and **J** Cell invasion assay after co-transfection of *STOX2* cDNA or miR-30a inhibitor and *STOX2* siRNA into HTR-8 cells. Bar = 100 μm. *P < 0 .05 or **P < 0 .01, ***P < 0.001

We investigated the functions of STOX2 and miR-30a in trophoblastic cells based on our previous research, which showed that increasing miR-30a levels could inhibit the proliferation and metastasis abilities of JAR and HTR-8 cells. We performed CCK-8 assays (Figs. 4A, B; 5A, B), colony formation assays (Figs. 4C, D; 5C, D), and EdU assays (Figs. 4E, F; 5E, F) to explore cell proliferation. The results showed that overexpression of miR-30a and *STOX2* could increase the proliferation of cells compared with that in the miR-30a mimics group. Conversely, in cells transfected with STOX2 siRNA, the opposite results were obtained. Transwell migration and invasion assays revealed that enhanced expression of *STOX2* impaired the ability of the miR-30a mimics to decrease trophoblastic cell metastasis (Fig. 4G, I; 5G, I). Compared with the miR-30a inhibitor group, co-transfection with the *STOX2* siRNA and the miR-30a inhibitor also blocked migration and invasion (Fig. 4H, J; 5H, J). These data suggest that STOX2 increased the proliferation and metastasis of JAR and HTR-8 cells.

### miR-30a influences the ERK 1/2, AKT, and p38 signaling pathways by regulating the expression of *STOX2* in trophoblastic cells

**Fig. 6:**
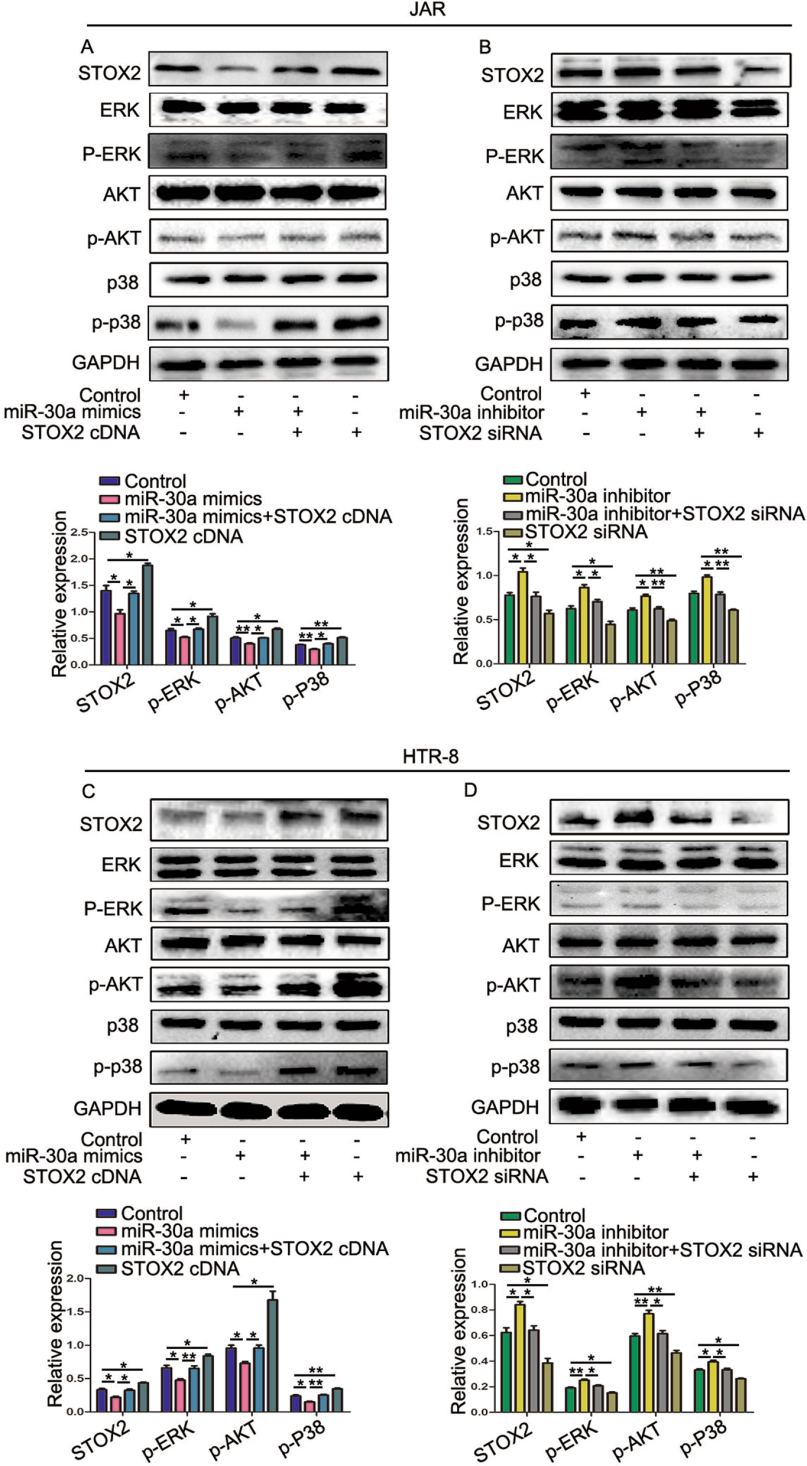
miR-30a reduces *STOX2* expression, which affects ERK 1/2, AKT, and p38 signaling pathways in trophoblastic cells. **A** and **B** Protein levels of ERK, AKT, and P38 signaling pathways-related proteins in cells co-transfected miR-30a mimics and *STOX2* cDNA, and comparison of the levels of p-ERK1/2, p-AKT, and p-P38 in each group. **C** and **D** The protein levels of ERK, AKT, and P38 signaling pathways-related proteins in cells co-transfected the miR-30a inhibitor and *STOX2* siRNA, and comparison of the levels of p-ERK1/2, p-AKT, and p-P38 in each group. *P < 0 .05 or **P < 0 .01, ***P < 0.001

We found that miR-30a and STOX2 mediated trophoblastic cell proliferation and metastasis in vitro. We next studied the molecular mechanisms by which miR-30a and STOX2 inhibit the growth, invasiveness, and migration of JAR and HTR-8 cells. After transfection of miR-30a mimics, the levels of phosphorylated AKT, ERK, and P38 decreased and but the levels of total AKT, ERK, and P38 did not change. Conversely, upregulation of *STOX2* increased the levels of p-AKT, p-ERK, and p-P38. Cotreatment with miR-30a mimics and *STOX2* cDNA further increased the levels of p-AKT, p-ERK, p-P38, without any changes in the total protein levels of AKT, ERK, and P38 (Fig. 6A, C). Similar levels of AKT, ERK, and P38 proteins were observed in miR-30a inhibitor and STOX2 siRNA co-transfected group, miR-30a inhibitor group, STOX2 siRNA group, and NC-transfected group. *STOX2* siRNA transfection reduced the levels of p-AKT, p-ERK, p-P38 in the presence of miR-30a inhibitor in JAR and HTR-8 cells (Fig. 6B, D). These results suggested that miR-30a targets *STOX2*, which affects the AKT/p-AKT, ERK/p-ERK, P38/p-P38 signaling pathways, resulting in inhibited cell proliferation and metastases. However, further experiments are required to validate this hypothesis.

## Discussion

The hydatidiform mole is one of the most common GTDs, with a prevalence of 1:1000 in North America and Europe; however, it is more common in South America and Asia. Differences in histology, genetics, and clinical features allow hydatidiform moles to be divided into complete moles (CHM) and partial moles (PHM) [32, 33]. Ultrasound is the main method for the clinical detection of a hydatidiform mole; however, it is also determined by the age of the fetus. Worldwide, hydatidiform moles are usually diagnosed at an early stage; however, in developing countries, patients are still diagnosed in early pregnancy with complications [34]. Although hydatidiform moles are benign, there is still a high possibility of developing malignant trophoblastic tumors. Clinical treatment for hydatidiform moles comprises curettage and hysterectomy. In the later stage, the content of human chorionic gonadotropin (hCG) should be detected to prevent recurrence. Hydatidiform moles and their curative surgery not only cause gross physical damage to patients, but also induce a large psychological burden. Therefore, new treatment methods are urgently needed.

miRNAs regulate many genes and are involved in the development of a variety of tumors [35, 36]. miR-30a plays a key role in many types of human cancers, with anti-cancer effects in non-small cell carcinoma [37], breast tumors [38], renal cell carcinoma [39], and colorectal cancer [40]. miR-30a prevents DNA replication and leads to DNA degradation by targeting *RPA1* (encoding replication protein A1), which induces P53 expression, and triggers S-phase checkpoints, preventing cell cycle progression, and ultimately leading to cancer cell death [41]. In addition, miR-30a regulates *EYA2* (encoding EYA transcriptional coactivator and phosphatase 2) to mediate the G1/S cell cycle and the expression of related cyclins [42]. In gallbladder cancer, miR-30a could directly target *E2F7* (encoding E2F transcription factor 7) to regulate epithelial-mesenchyme transition (EMT) and metastasis, and participated in cancer progression [43]. The expression of miR-30a-3p was significantly increased in the preeclamptic placenta tissue, and regulated trophoblast invasion and apoptosis by targeting *IGF-1* [23]. In the present study, we found that downregulation of miR-30a enhanced the proliferation, migration, and invasion abilities of trophoblastic cells, and had lower expression in hydatidiform mole tissues than in the normal placenta, which suggested that miR-30a is involved in the development of hydatidiform moles.

STOX2 is considered to be the only other member of the family that includes STOX1; to date, its function has been unclear. However, studies on STOX1 have reported that the polyploidy defect that appears before incomplete invasion of extravillous trophoblasts caused by STOX1 dysfunction seems to be the center of pre-eclampsia, and activates phosphatidylinositol-4,5-bisphosphate 3-kinase (PI3K)/AKT/forkhead box (FOX) signaling pathway [24]. It has been reported that STOX1A regulates the cell cycle by binding to cyclin B1 to regulate mitosis [44]. An increase in the level IGF1 led to an increase in the expression of STOX1 in extravillous trophoblasts through the mitogen activated protein kinase (MAPK) pathway, thereby identifying a new signaling cascade involved in maternal–fetal communication [45]. Homologous genes have similar biological functions. The RNA encoded by intron 3 (IT3) of *STOX2* can affect the alternative splicing of host genes in placental cells. The long noncoding RNA *STOX2-IT3* affects genes involved in trophoblast differentiation and invasion, thus affecting the pathogenesis of eclampsia [46]. In the present study, we found that STOX2 not only reduced cell proliferation and metastasis, but also was highly expressed in hydatidiform mole tissues, which suggested that STOX2 might induce hydatidiform moles.

The biological role of miRNAs is achieved by regulating downstream target genes. Our results revealed that *STOX2* was the direct target gene of miR-30a. Moreover, we found that *STOX2* mRNA levels were reduced by upregulating miR-30a in trophoblastic cells. We also detected that downregulation of *STOX2* could impair the proliferation and metastasis abilities of trophoblastic cells. However, the molecular mechanism by which miR-30 regulates the biological role of STOX2 is not clear. The ERK, AKT, and P38 signaling pathways are important for tumor proliferation and metastasis, and are involved in the pathogenesis of various cancers. Studies have shown that miR-30a-5p targeting *NEUROD1* (encoding neuronal differentiation 1) could improve inflammatory responses and oxidative stress through the MAPK/ERK signaling pathway in cases of spinal cord injury [47]. miR-30a released by the p53 r273h mutation can inhibit the expression of *IGF1R* (encoding insulin like growth factor 1 receptor), which leads to the activation of IGF-1-r-AKT signal cascade in tumor cells [48]. Moreover, mir-30a was downregulated significantly in highly metastatic colorectal cancer cell lines and metastatic tissues, and its mechanism involves regulation of the AKT/mechanistic target of rapamycin (mTOR) signaling pathway by targeting *PIK3CD* (encoding PI3K catalytic subunit delta) expression, thereby regulating the metastasis of cancer cells [49]. In addition, the mir-30-5p-transcription factor 21 (TCF21)-MAPK/P38 signaling pathway might be a potential biomarker or therapeutic target of atherosclerosis [50]. Interestingly, in our study, we demonstrated that overexpression of miR-30a inhibited the phosphorylation of ERK, AKT, and P38, whereas upregulation *STOX2* ameliorated this suppressive effect. These data further confirmed that the aggressive proliferation potential of hydatidiform moles was caused, at least in part, by the low expression of miR-30a, which regulates *STOX2* to activate the ERK, AKT, and P38 signaling pathways. Further research into the specific molecular mechanism of this process is required, which could lead to new treatment strategies for hydatidiform moles by targeting miR-30a and STOX2.

## Conclusions

In the summary, we demonstrated that STOX2 is highly regulated in hydatidiform moles. miR-30a could suppress the proliferation and invasion ability of trophoblastic cells through targeting *STOX2*, which affected the AKT, ERK, and P38 signaling pathways. Our findings offer insights into the mechanism of hydatidiform mole formation. STOX2 and miR-30a could be developed as a biomarkers and therapeutic targets for hydatidiform moles in the future.

## Supplementary Information

**Additional file 1: Figure S1.** The effect of miR-30a on the cell cycle and proliferation. (A) A colony-formation assay was used to test the proliferation of HTR-8 cells after transfection with miR-30a mimics/negative control or miR-30a inhibitor/negative control. (B) The phase population was compared with control transfectants using flow cytometry in Ishikawa cells after transfection with miR-30a mimics and miR-30a inhibitor.

**Additional file 1: Figure S2.** Correlation coefficient between miR30a and STOX2 expression.

## Data Availability

The datasets generated and analyzed during the current study are available from the corresponding author on reasonable request.
