# Supplementary figures and images for: miR-30a targets STOX2 to increase cell proliferation and metastasis in hydatidiform moles via ERK, AKT, and P38 signaling pathways
Source: Cancer Cell Int. 2022 Mar 4;22:103. doi: 10.1186/s12935-022-02503-3 (PMC8895545; doi:10.1186/s12935-022-02503-3)

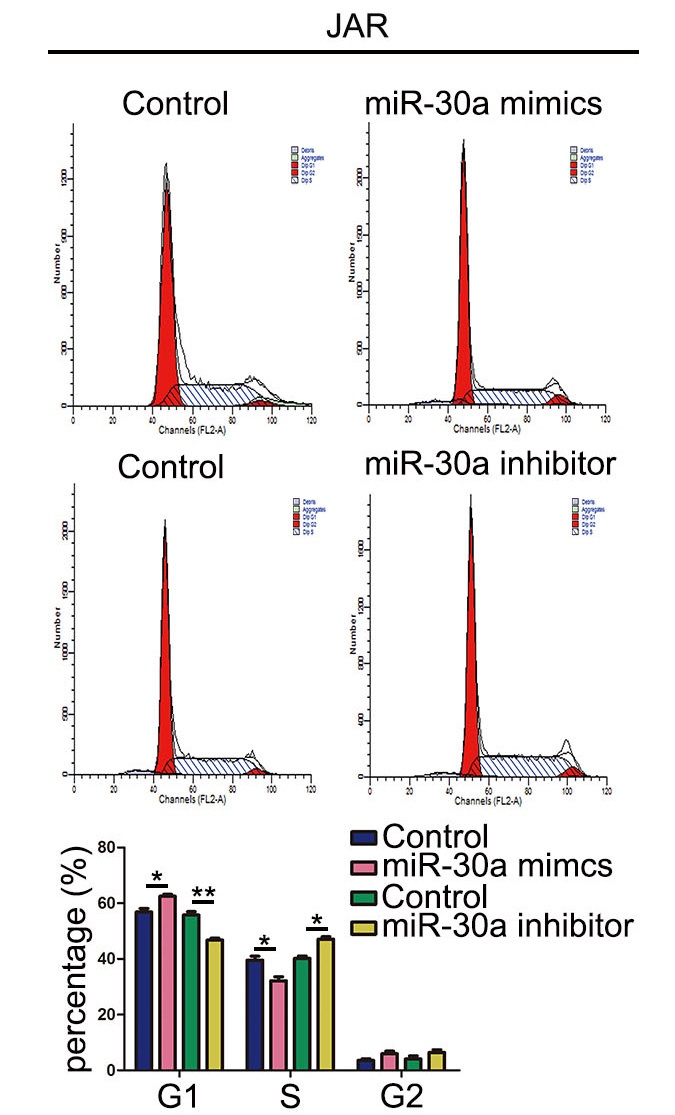

Supplement: Supplementary file 1 — Additional file 1: Figure S1. The effect of miR-30a on the cell cycle and proliferation. (A) A colony-formation assay was used to test the proliferation of HTR-8 cells after transfection with miR-30a mimics/negative control or miR-30a inhibitor/negative control. (B) The phase population was compared with control transfectants using flow cytometry in Ishikawa cells after transfection with miR-30a mimics and miR-30a inhibitor. [file 12935_2022_2503_MOESM1_ESM.jpg]

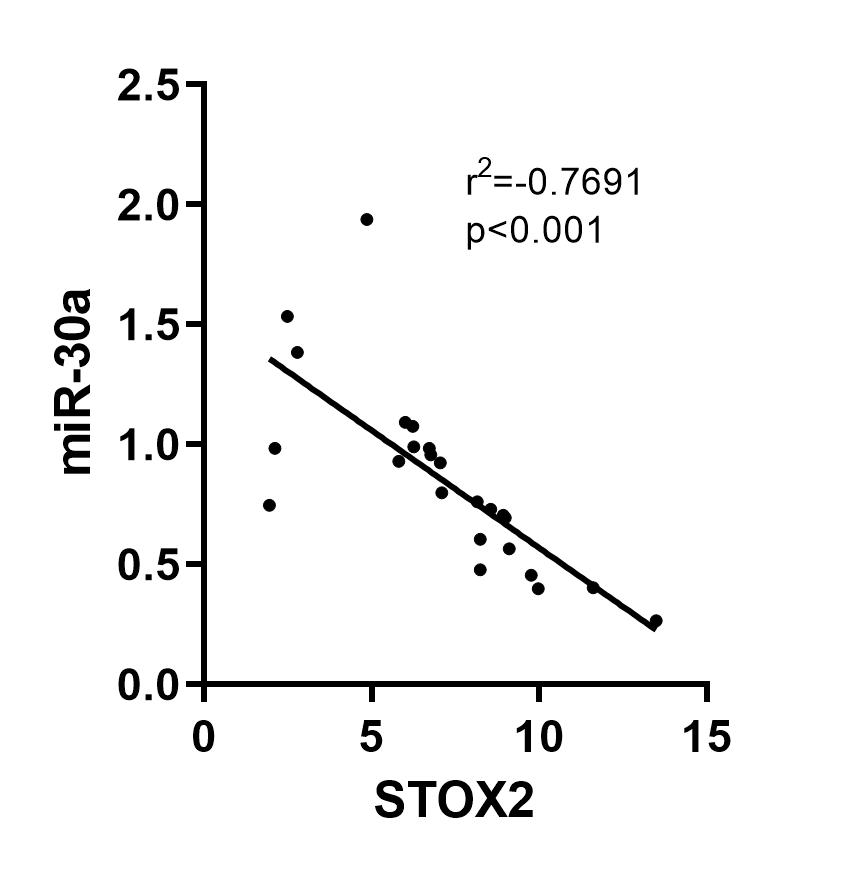

Supplement: Supplementary file 2 — Additional file 1: Figure S2. Correlation coefficient between miR30a and STOX2 expression. [file 12935_2022_2503_MOESM2_ESM.jpg]
